# Seed maturation associated transcriptional programs and regulatory networks underlying genotypic difference in seed dormancy and size/weight in wheat (*Triticum aestivum* L.)

**DOI:** 10.1186/s12870-017-1104-5

**Published:** 2017-09-16

**Authors:** Yuji Yamasaki, Feng Gao, Mark C. Jordan, Belay T. Ayele

**Affiliations:** 1 Department of Plant Science, University of Manitoba, 222 Agriculture Building, Winnipeg, MB R3T 2N2 Canada; 2 Morden Research and Development Centre, Agriculture and Agri-Food Canada, Morden, MB R6M 1Y5 Canada

**Keywords:** Embryo, Endosperm, Genotype, Seed maturation, Transcriptome, *Triticum aestivum*, Wheat

## Abstract

**Background:**

Maturation forms one of the critical seed developmental phases and it is characterized mainly by programmed cell death, dormancy and desiccation, however, the transcriptional programs and regulatory networks underlying acquisition of dormancy and deposition of storage reserves during the maturation phase of seed development are poorly understood in wheat. The present study performed comparative spatiotemporal transcriptomic analysis of seed maturation in two wheat genotypes with contrasting seed weight/size and dormancy phenotype.

**Results:**

The embryo and endosperm tissues of maturing seeds appeared to exhibit genotype-specific temporal shifts in gene expression profile that might contribute to the seed phenotypic variations. Functional annotations of gene clusters suggest that the two tissues exhibit distinct but genotypically overlapping molecular functions. Motif enrichment predicts genotypically distinct abscisic acid (ABA) and gibberellin (GA) regulated transcriptional networks contribute to the contrasting seed weight/size and dormancy phenotypes between the two genotypes. While other ABA responsive element (ABRE) motifs are enriched in both genotypes, the prevalence of G-box-like motif specifically in tissues of the dormant genotype suggests distinct ABA mediated transcriptional mechanisms control the establishment of dormancy during seed maturation. In agreement with this, the bZIP transcription factors that co-express with ABRE enriched embryonic genes differ with genotype. The enrichment of SITEIIATCYTC motif specifically in embryo clusters of maturing seeds irrespective of genotype predicts a tissue specific role for the respective TCP transcription factors with no or minimal contribution to the variations in seed dormancy.

**Conclusion:**

The results of this study advance our understanding of the seed maturation associated molecular mechanisms underlying variation in dormancy and weight/size in wheat seeds, which is a critical step towards the designing of molecular strategies for enhancing seed yield and quality.

**Electronic supplementary material:**

The online version of this article (10.1186/s12870-017-1104-5) contains supplementary material, which is available to authorized users.

## Background

Wheat (*Triticum aestivum* L.) is one of the most economically important cereal crops in the world, and its seeds serve as basic units of propagation, and source of food, feed and raw material for a wide-range of industrial products. Owing to all these agronomic and economic importance, understanding the genetic and molecular mechanisms regulating wheat seed developmental programs, and thereby yield and quality has been the subject of recent wheat genomic studies. In cereals such as wheat, seed development starts with fertilization events and terminates with the formation of mature quiescent seeds, and the entire developmental process is comprised of three phases [1, 2]. The first phase, which is also referred to as early phase, comprises double fertilization that forms the embryo and endosperm, syncytium formation and endosperm cellularization events. The second phase is characterized by differentiation, which includes events associated with the formation of different cell types, endoreduplication and deposition of storage reserves. Maturation forms the third phase of seed development and it is characterized mainly by programmed cell death, dormancy and desiccation. The degree of dormancy induced during seed maturation is tightly associated with the level of tolerance to preharvest sprouting, which is defined as the germination of seeds on the spike prior to harvest, that causes significant losses in seed yield and quality. Dormancy is regulated by genetic and environmental factors, and the plant hormones abscisic acid (ABA) and gibberellin (GA) are reported to have important roles in the induction, maintenance and release of dormancy. Another seed related trait of economic importance is seed size/weight that forms one of the major components of grain yield. It is determined by deposition of storage reserves that occurs following histodifferentiation through to physiological maturity [3]. Developing wheat grains appear to attain physiological maturity at about 42 DAA and harvest maturity by 1–2 weeks thereafter [4, 5].

Transcriptomic studies in different plant systems have led to the identification of transcriptional programs and regulatory networks underlying molecular functions associated with cellular activities. In this context, seed development in the model plant Arabidopsis has been shown to be characterized by distinct and overlapping functional identities and regulatory networks in different regions and sub-regions of seeds [6]. For example, genes upregulated specifically in the embryos and endosperms of mature seeds are enriched with ABA responsive element (ABRE) motif, which acts as a binding site for bZIP transcription factors (TFs), and molecular functions associated with ABA stimulus and oil biogenesis. Furthermore, seed specific TFs with unknown and known roles including LEAFY COTYLEDON and FUSCA3 have been implicated in mediating the regulatory networks required for programming seed development [7]. Transcriptomic analysis of developing seed tissues in cereals such as maize, rice and barley have also led to the identification of genes involved in the programming of seed developmental and maturation processes, and elucidation of the underlying functional transitions [8–10]. Furthermore, different tissue types of developing seeds of cereals such as embryo and endosperm have been shown to exhibit distinct gene expression profiles and therefore molecular functions [9], suggesting the ditinct roles they play in regulating seed traits. For example, genes involved in the synthesis and signaling of ABA, which influences starch biosynthesis in the endosperm and desiccation tolerance in the embryo, differ with tissue types.

Similarly, large scale gene expression studies of developing wheat seeds have provided important insights into the developmental shifts in gene expression, distinct and overlapping transcriptional reprogramming between different tissues such as aleurone and endosperm, and cell-type and stage-dependent transcriptional dynamics and genome interplays [11–13]. However, these studies analyzed either the whole seed, or different tissue types only during the early and differentiation/grain filling phases of seed development. Previous studies also investigated the transcriptome of wheat seed, for example, with respect to dormancy [14], however, most of the dormancy studies have been focused on post-harvest of seeds. As a result, the spatiotemporal transcriptional programs and regulatory networks underlying the establishment and maintenance of dormancy during the maturation phase of wheat seed development are poorly understood. To this effect, this study performed comparative transcriptomic analysis of the endosperm and embryo of maturing seeds between two wheat genotypes, AC Domain and RL4452, characterized by contrasting degree of dormancy at four developmental stages (20–50 DAA) that are critical to the acquisition of dormancy and desiccation tolerance. Owing to their difference in seed dormancy, these genotypes have been used as parental lines to generate breeding populations and thereby map QTLs associated with tolerance to preharvest sprouting [15, 16]. Since the two genotypes also exhibit contrasting final seed size/weight and represent different genetic materials than those studied previously, our transcriptomic analysis was also aimed at elucidating molecular features that regulate deposition of storage reserves during the seed maturation phase.

## Result and discussion

### Comparative analysis of seed phenotypes during and after maturation

**Fig. 1:**
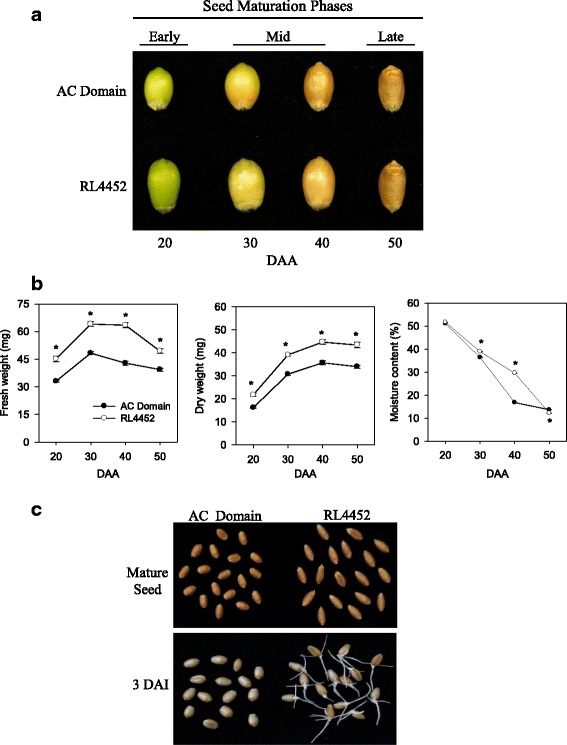
Maturing seeds of wheat. Seeds of AC Domain and RL4452 genotypes from 20 to 50 DAA (**a**); changes in seed fresh weight, dry weight and moisture content during maturation - data are means ± SE (*n* = 20–23) and asterisks indicate statistically significant difference in seed fresh weight, dry weight and moisture content between the two genotypes (*P* < 0.05; t-Student test) (**b**); and difference in seed size and dormancy/germination phenotypes between mature seeds of the two genotypes (**c**)

Seed maturation in both AC Domain and RL4452 genotypes was studied from 20 to 50 DAA (Fig. 1a). The seeds of both genotypes showed increases in fresh and dry weights from 20 to 30 DAA, and after 30 DAA the fresh weights starts to decline in both genotypes, while the dry weights increased slightly from 30 to 40 DAA and remained constant thereafter (Fig. 1b) as observed before [17]. Overall, the RL4452 seeds exhibited consistently significantly higher fresh and dry weights over the entire seed maturation period than that of AC Domain, suggesting more storage reserves deposition in RL4452 seeds and therefore production of larger seeds. The two genotypes exhibited similar rate of water loss from 20 to 30 DAA (Fig. 1b). However, AC Domain seeds desiccated at a higher rate from 30 to 40 DAA (2% moisture loss/day for AC Domain vs. ~1% moisture loss/day for RL4452) while the RL4452 seeds desiccated at a higher rate from 40 to 50 DAA (1.7% moisture loss/day for RL4452 vs. 0.3% moisture loss/day for AC Domain). The overall desiccation rate appeared to be higher in RL4452 seeds (1.32% moisture loss/day for RL4452 vs. 1.25% moisture loss/day for AC Domain), leading to seed moisture contents at 50 DAA of 13.6% and 12.2% in AC Domain and RL4452, respectively. Therefore, the time points studied here represent the stage from late reserve accumulation to desiccation, and we divided this period into early (20 DAA), mid (30–40 DAA) and late (50 DAA) phases of seed maturation (Fig. 1a). Freshly harvested mature seeds of RL4452 exhibited over 93% germination within 24 h imbibition, indicating that they are non-dormant, while only 15% of the corresponding AC Domain seeds germinated even after 5-day imbibition, indicating their dormant phenotype [18, 19] (Fig. 1c).

### Genotype- and tissue-specific gene expression profiles during wheat seed maturation

**Fig. 2:**
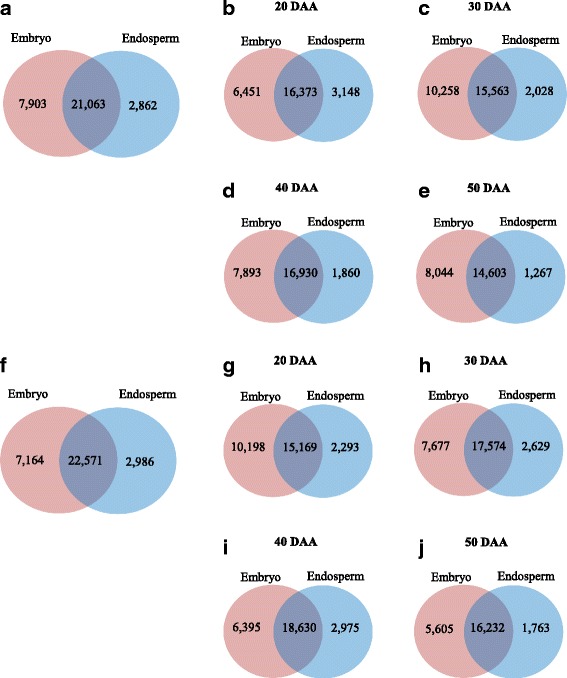
Number of probesets expressed in the embryo and/or endosperm during seed maturation. Probesets expressed at one or more time points (**a**, **f**) and at each time point (**b**–**e**, **g**–**j**) in the tissues of AC Domain (**a**–**e**) and RL4452 (**f**–**j**) were determined using MAS5.0 (*P* < 0.05)

It appeared from our analysis that more probesets are expressed in the embryo and/or endosperm tissues at least at one time point during seed maturation in RL4452 (32,721 probesets; 53.4% of the probesets on the Wheat GeneChip) than in AC Domain (31,828 probesets; 51.9% of the probesets on the Wheat GeneChip) (Fig. 2), suggesting the prevalence of more transcriptional activity in maturing seeds of RL4452, a genotype that produces larger seeds (on fresh and dry weight basis). The number of probesets expressed in the embryo of each genotype overall or at each stage of development was more than that observed in the endosperm (Fig. 2), suggesting that the two tissue types of maturing wheat seeds are characterized by differing gene transcriptional states. Scatter-plot expression analysis revealed high reproducibility between replicates of each sample (r^2^ > 0.95), and a strong correlation was observed between the microarray and qPCR data for 10 randomly selected differentially expressed probesets (Additional file 1: Fig. S1).

#### Temporal shifts in gene expression profiles during seed maturation varies with genotype and tissue

**Fig. 3:**
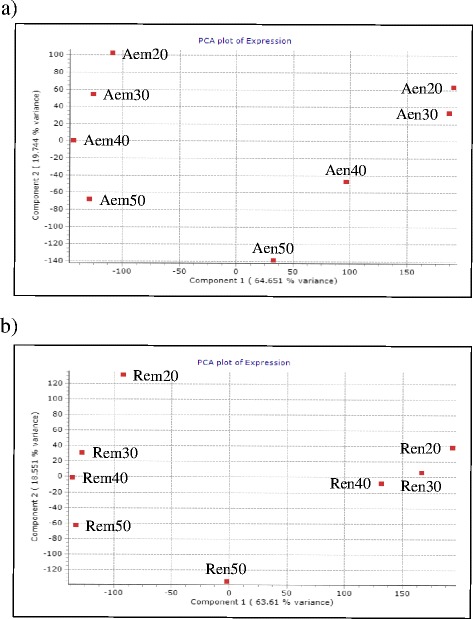
Principal component analysis of seed maturation transcripts. Embryo and endosperm of maturing AC Domain (**a**) and RL4452 (**b**) seeds. Aem, AC Domain embryo; Aen, AC Domain endosperm; Rem, RL4452 embryo; Ren, RL4452 endosperm at 20, 30, 40 and 50 days after anthesis. Principal components (PC)1 representing tissue identity (64.65% in AC Domain and 63.61% in RL4452) and PC2 representing developmental stages (19.74% in AC Domain and 18.55% in RL4452) with a cumulative percentage > 80% in each genotype

Principal component analysis (PCA) of the transcriptomic data revealed three different expression patterns for the endosperm of AC Domain; the 20 and 30 DAA endosperms are more closely related while each of those at 40 DAA and 50 DAA represent a separate and distinct group, indicating deviation in gene expression as maturation proceeds from the mid to late phase (Fig. 3a). In RL4452, the endosperm at the early to mid-phases (20 to 40 DAA) of seed maturation appeared to have a close relationship while those at the late phase (50 DAA) represent a distinct group, indicating that a major shift in gene expression occurs only as the seed desiccates (Fig. 3b). The two genotypes differ in seed size/weight, which is determined mainly by the deposition of storage reserves in the endosperm that has been shown to express genes involved in carbohydrate metabolic processes [11]. It is therefore likely that the differential shifts in endospermic gene expression between the two genotypes might contribute to the difference in seed size/weight. Embryos of AC Domain at each time point of seed maturation appeared to represent a distinct group, implying a constant shift in gene expression profile during the seed maturation phase. In RL4452, however, embryos at the mid phase (30 and 40 DAA) appeared to be closely related while each of those at the early (20 DAA) and late (50 DAA) phases formed a distinct group, suggesting that major shifts in gene expression occur as seeds transitioned from early to mid and from mid to late phases of maturation. Given that the embryo can represents a major component of seed dormancy [20], the distinct gene expression shift observed in the developing AC Domain embryos might underlie the induction of dormancy exhibited by the mature seeds of this genotype [18, 19] (Fig. 1c). Our data overall suggest that the genotype specific temporal shift in gene expression profile thereby molecular function in each of the endosperm and embryo tissues of maturing wheat seeds represent the variations in seed size/weight and dormancy level.

#### Tissue types of maturing seeds are characterized by distinct but temporally and genotypically overlapping gene expression profiles

Our PCA analysis also showed larger difference in gene expression pattern between the endosperm and embryo tissues mainly at the early- and mid-phases of seed maturation irrespective of genotype, suggesting that the two tissues are characterized by distinct molecular functions. However, as the seed enters into the late phase of maturation the difference in gene expression profile between the two tissues became smaller in both genotypes but slightly more pronounced in RL4452, which exhibited a more rapid rate of desiccation during transition from mid to late phases, suggesting desiccation induced repression of gene transcription [21, 22]. Likewise, difference in gene expression profile between the endosperm and embryo tissues have been shown to shrink during the late phases of seed maturation in maize [8].

Hierarchical clustering of the embryo and endosperm tissues also revealed overlap between the embryo samples and 50 DAA endosperm in RL4452 while the clustering in AC Domain is based primarily on tissue identity (Additional file 2: Fig. S2).

### Genotype and tissue specific co-expression clusters during wheat seed maturation

**Fig. 4:**
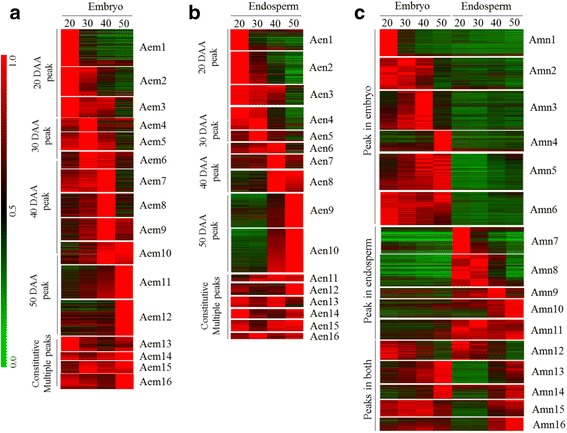
Co-expression gene clusters of AC Domain (A). All probesets expressed in the embryo (**a**), endosperm (**b**) or both (**c**) at one or more time points were clustered based on the peak expression at a given time point(s) during seed maturation. The expression level of probesets in each of the embryo (em) (**a**), endosperm (en) (**b**) or both (mn) (**c**) tissues was determined relative to a time point with the highest RMA-normalized signal intensity, which was arbitrarily set to a value of 1 and represented by red color in the red-green scale shown on the left side of the heat map

**Fig. 5:**
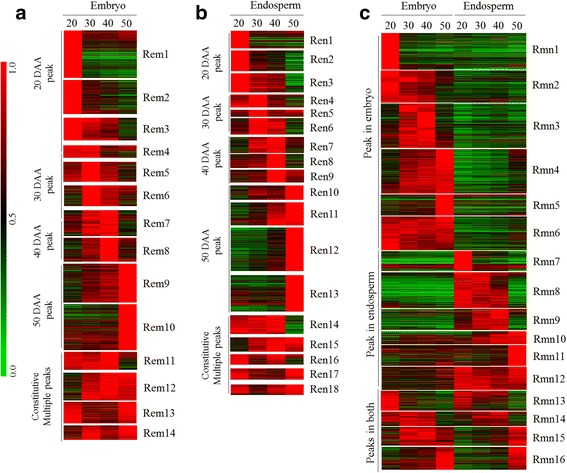
Co-expression gene clusters of RL4452 (R). Clustering of probesets expressed in the embryo (em) (**a**), endosperm (en) (**b**) or both (mn) (**c**) at one or more time points. Figure descriptions are as shown in Fig. 4

To better understand the commonalities and differences in transcriptional programs underlying variation in seed size/weight and dormancy between the two genotypes, we performed cluster analysis of all probesets expressed at least at one time point of seed maturation in each of the embryo (28,966 probesets in AC Domain; 29,735 probesets in RL4452) and endosperm (23,925 probesets in AC Domain; 25,557 probesets in RL4452) tissues (Figs. 4 and 5). To confirm tissue specificity of the probesets, further cluster analysis was performed by combining those expressed in both embryo and endosperm (31,828 probesets in AC Domain; 32,721 probesets in RL4452). Accordingly, we generated 16 clusters for each of the embryo, endosperm, and embryo and endosperm categories of AC Domain; and 14, 18 and 16 clusters for each of the embryo, endosperm, and embryo and endosperm categories of RL4452, respectively (Figs. 4 and 5, Additional file 7: Table S1); promoter motif enrichment was used as a criterion to determine the total number of clusters per category. The embryo or endosperm clusters in both genotypes exhibited peak expression at one or more time points during seed maturation (Figs. 4a,b and 5a,b) while gene clusters generated by combining the embryo and endosperm are roughly divided into three categories in both genotypes (Figs. 4c and 5c). The first category represents clusters with peak expression at least at one time point specifically in the embryo, the second category represents clusters with peak expression at least at one time point specifically in the endosperm, and the third category represents clusters with peak expression at least at one time point in both embryo and endosperm tissues.

#### Functional identity of genotypically and temporally overlapping embryo-specific clusters

**Fig. 6:**
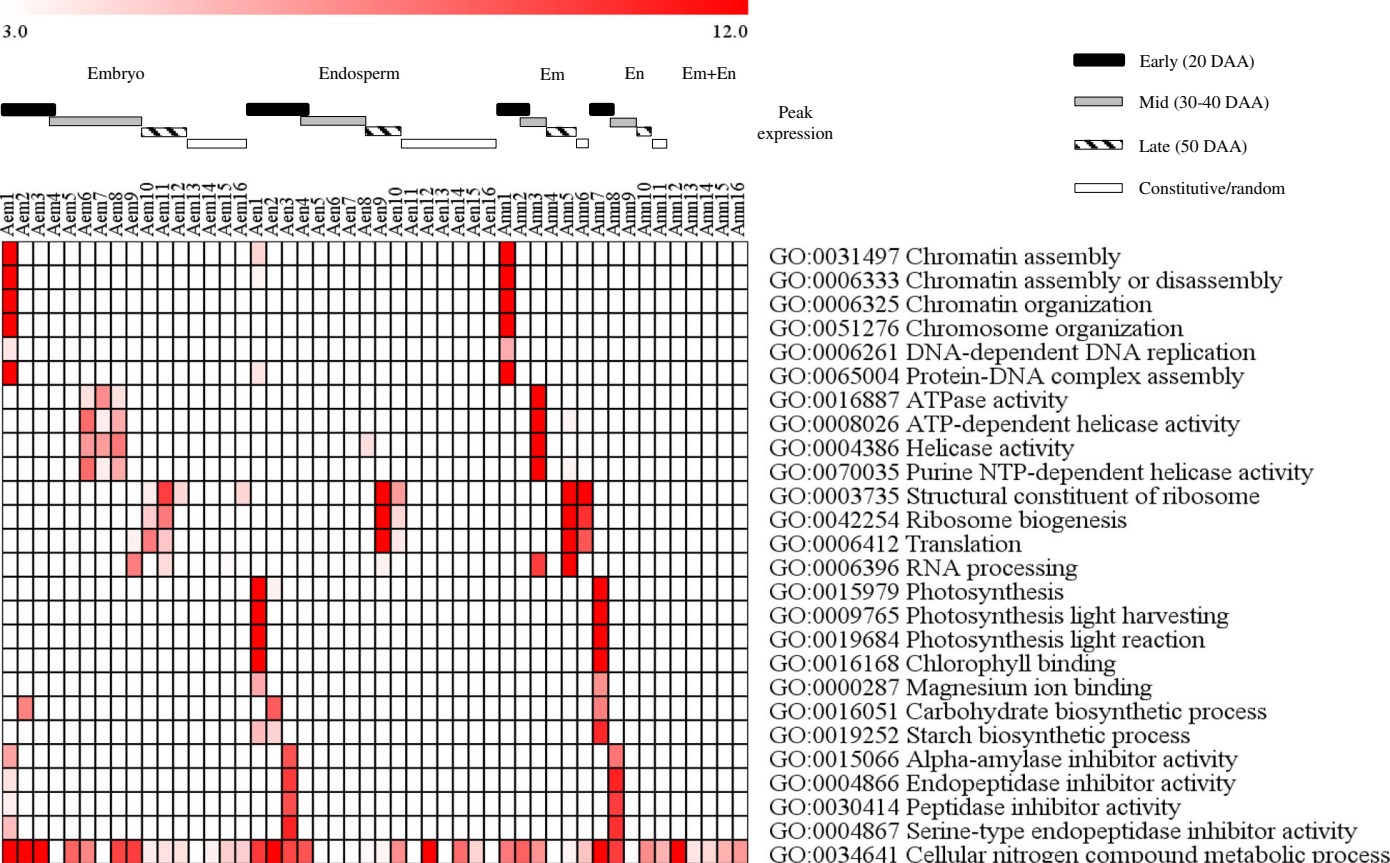
Gene ontology enrichment in the co-expression clusters of maturing AC Domain (A) seeds. The GO terms listed are consistently enriched (*P* < 10e^−3^) in the embryo (em) or endosperm (en) clusters and the corresponding clusters of both (mn) tissues; *P* value is shown by the color scale at the top (1/log10). Enriched GO terms and the respective *P* values can be found in Additional file 8: Table S2. Black, grey and striped bars represent clusters with peak expression at early (20 DAA), mid (30 and 40 DAA) and late (50 DAA) phases of seed maturation, respectively. The white bar represents gene clusters with constitutive/random expression patterns

**Fig. 7:**
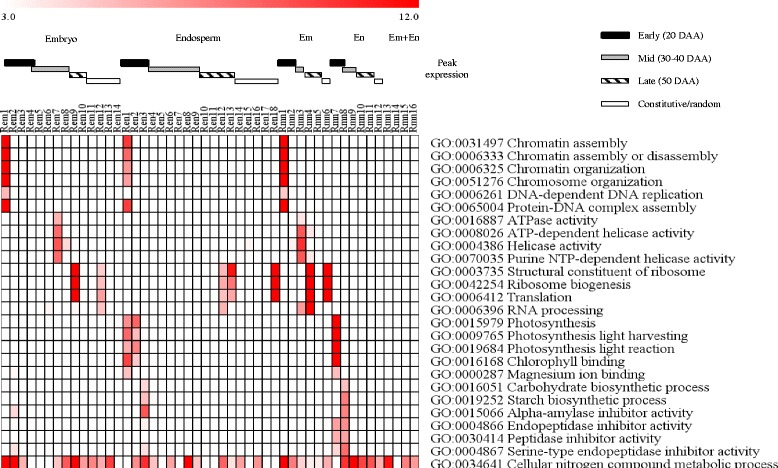
Gene ontology enrichment in the co-expression clusters of maturing RL4452 (R) seeds. Figure descriptions are as shown in Fig. 6

To gain insights into distinct and overlapping tissue specific biological processes/molecular functions, gene ontology (GO) terms enriched in the embryo and endosperm gene clusters of both genotypes were identified (Figs. 6 and 7, Additional file 8: Table S2). Embryo clusters characterized by peak expression at the early phase with strong repression in the subsequent phases of seed maturation irrespective of genotype (Aem1 and Amn1; Rem1 and Rmn1) are enriched with chromatin/chromosome organization (GO:0031497, GO:0006333, GO:0006325, GO:0051276; *P* = 2.4e^−31^ ~ 3.10e^−18^), DNA-dependent DNA replication (GO:0006261; *P* = 1.1e^−6^ ~ 1.2e^−4^) and protein-DNA complex assembly (GO:0065004; *P* = 1.1e^−32^ ~ 8.9e^−21^) GO terms. Given that genes in these GO terms such as those encoding histone proteins (H2A/H2B/H3/H4) and DNA polymerases are involved in cellular proliferation [23], it is likely that their transcriptional activation at the early phase of maturation forms a mechanism underlying rapid growth of embryos and initiation of axial growth, which has been shown to occur during 15 to 30 DAA in wheat seeds [24] with no major role in the regulation of dormancy. Embryo clusters exhibiting peak expression during the mid-phase of seed maturation in both genotypes (Aem6–8 and Amn3; Rem7 and Rmn3) are enriched with ATPase activity (GO:0016887; *P* = 3.7e^−16^ ~ 1.2e^−4^) and helicase activity (GO:0008026, GO:0004386, GO:0070035; *P* = 5.3e^−24^ ~ 3.1e^−7^) GO terms that include genes encoding RNA helicase, SNF2 type helicase and ABC transporter proteins; and many of the RNA helicase probesets are annotated as DEAD-box type (Fig. 6, Additional file 8: Table S2). Given that the DEAD-box type RNA helicases are implicated in the regulation of growth and developmental events, and participate in the control of stress responsive genes [25, 26] and ABA biosynthesis [27], it is likely that the genes in these clusters form an integral part of a mechanism regulating embryo growth and desiccation-induced stress tolerance but not the induction of dormancy in maturing seeds.

Ribosome biogenesis and translation GO terms (GO:0003735, GO:0042254, GO:0006412; *P* = 2.2e^−61^ ~ 2.8e^−4^) are prevalent in embryo clusters of both genotypes exhibiting peak expression at mid to late phases of seed maturation (Aem10, Aem11, Amn5 and Amn6; Rem9, Rem12, Rmn4 and Rmn6). Furthermore, the RNA processing GO term (GO:0006396; *P* = 2.8e^−14^ ~ 4.3e^−4^), which includes RNA splicing and exosome genes, is enriched in embryo clusters exhibiting peak expression around the same time (Aem9, Aem11, Amn3 and Amn5; Rem6, Rem9, Rem12, Rmn3 and Rmn4). These results suggest the ability of the embryo in the mid to late phases of seed maturation to employ post-transcriptional regulatory mechanisms for the synthesis of maturation/desiccation associated proteins from extant transcripts [28, 29]. Alternatively, the results might imply maturation-mediated storage of translational transcripts in the seeds to help with the synthesis of proteins required for germination upon imbibition of non-dormant seeds [30]. In agreement with this hypothesis, the transcripts of ribosomal proteins are among the most abundant mRNAs stored in mature dry seeds of barley, and these genes exhibit transcriptional induction during imbibition of non-dormant seeds [31].

#### Functional identity of genotypically and temporally overlapping endosperm-specific clusters

The endosperm gene clusters of both genotypes with peak expression at the early phase of seed maturation (Aen1 and Amn7; Ren1 and Rmn7) are enriched with photosynthesis (GO:0015979, GO:0009765, GO:0019684; *P* = 4.7e^−23^ ~ 9.3e^−7^), chlorophyll binding (GO:0016168; *P* = 4.4e^−20^ ~ 1.9e^−13^) and magnesium ion binding (GO:0000287; *P* = 1.30× e^−7^ ~ 6.50× e^−6^) GO categories. Given that immature seeds of wheat and other cereals have photosynthetically active pericarp [32], which is part of the endosperm in this study, it is likely that the genes in these clusters are exclusively expressed in the pericarp. However, the absence of differential expression pattern of these genes between the two genotypes might suggest their minimal role in inducing the variation in seed size/weight. Endospermic gene clusters of both genotypes whose expression is peaked at the early phase and remain enhanced during the mid-phase (Aen3 and Amn8; Ren3 and Rmn8) are enriched with alpha-amylase inhibitor activity (GO:0015066; *P* = 7.3e^−10^ ~ 3.6e^−8^) and endopeptidase inhibitor activity (GO:0004866, GO:0030414, GO:0004867; *P* = 1.7e^−11^ ~ 7.3e^−4^) GO terms, suggesting the significance of repressing starch degradation during the seed maturation process. Similar to that observed in the embryo, the cellular nitrogen compound metabolic process (GO:0034641; *P* = 8.6e^−27^) appeared to be enriched in several endosperm clusters of both genotypes exhibiting peak expressions at different time points. It is likely that these nitrogen metabolism genes are involved in the supply of nitrogen to the maturing endosperm but are less likely to contribute to the variation in seed size/weight. Owing to the enrichment of the same GO term in germinating wheat and barley seeds, the nitrogen metabolism genes have also been suggested to play a role of providing nitrogen to the growing embryo [33, 34]. No GO term enrichment is evident in endosperm specific clusters, as confirmed by combining probesets expressed in both tissues, of both genotypes exhibiting enhanced/peak expression during mid to late phase of maturation (Amn9–11; Rmn9–12), and this might be attributed to cellular death and desiccation/quiescence, leading to a pause or a decrease in some cellular metabolic activities [35].

### Gene regulatory networks during the maturation phase of wheat seed development

#### Genotypically distinct ABA regulated gene networks might contribute to the modulation of starch synthesis and thereby difference in seed weight and size

**Fig. 8:**
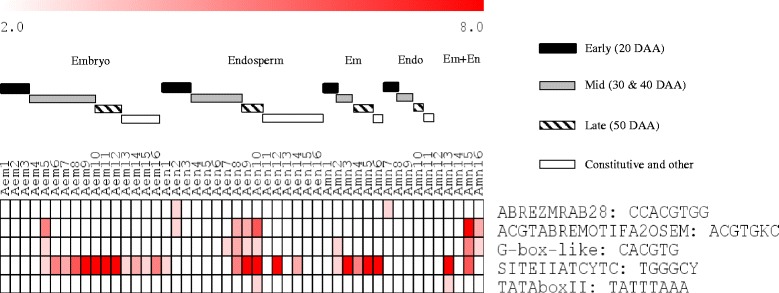
Promoter motif enrichment in the co-expression clusters of maturing AC Domain (A) seeds. Motif enrichments (*P* < 10e^−3^) are found in embryo (em) and endosperm (en) clusters corresponding to the same seed maturation phase or in tissue specific clusters; *P* value (1/log10) is shown by the color scale at the top. Enriched motifs and the respective *P* values can be found in Additional file 9: Table S3. Black, grey and striped bars represent clusters with peak expression at early (20 DAA), mid (30 and 40 DAA) and late (50 DAA) phases of seed maturation, respectively. The white bar represents gene clusters with constitutive/random expression patterns. Degenerate characters in the consensus sequence: K = G/T, S = C/G, R = G/A, Y = C/T

Endospermic gene clusters whose expression is peaked at early phase (20 DAA) in both genotypes, and maintained elevated level of expression in subsequent phases (30–40 DAA) only in RL4452 (Aen2 and Amn7, and Ren3 and Rmn8) are enriched with carbohydrate and starch biosynthetic processes (GO:0016051 and GO:0019252; *P* = 2.5e^−11^ ~ 4.0e^−5^). Previous studies have shown that the expression pattern of starch biosynthesis genes is closely associated with that of starch accumulation in the endosperm of developing wheat seeds [17, 36]. Thus, our data might suggest the maintenance of elevated starch deposition in the maturing endosperm of RL4452 that produces larger seeds than that of AC Domain (Fig. 1). However, comparative analysis of the kinetics of starch accumulation between the two genotypes is required to clarify this. Given that ABA has been implicated in reducing starch synthesis ability of developing wheat seeds [17, 37] and this role of ABA has been suggested to be mediated by SNF1 kinase [9], the prevalence of ABRE motif specifically in the AC Domain clusters (Aen2 and Amn7) (Fig. 8) might suggest increased ABA sensitivity and therefore decreased accumulation of starch.

#### Genotypically, temporally and spatially distinct ABA regulated gene networks might contribute to the modulation of ABA response and thereby difference in seed dormancy

**Fig. 9:**
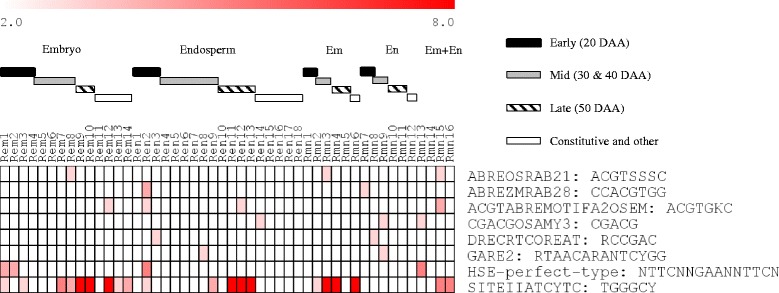
Promoter motif enrichment in the co-expression clusters of maturing RL4452 (R) seeds. Figure descriptions are as shown in Fig. 8

To predict gene regulatory networks underlying variation in seed size/weight and dormancy between the two genotypes, the 500 bp upstream region of rice homologs corresponding to the wheat probesets (*P* < 10e^−30^) in each cluster was analyzed for motif enrichment using Osiris promoter database [38] (Figs. 8 and 9, Additional file 9: Table S3); motif enrichment was limited to rice due to insufficient database support for other cereal crops. Our data showed that several ABRE motifs containing the core ACGT sequence are enriched in several embryo and endosperm clusters of maturing seeds in both genotypes including ABREOSRAB21 (ACGTSSSC), ABREZMRAB28 (CCACGTGG), ACGTABREMOTIFA2OSEM (ACGTGKC) and G-box-like (CACGTG), which act as binding sites for bZIP TFs such as ABI5 [39, 40]. In agreement with this result, genes highly expressed in mature seeds of Arabidopsis have been shown to be enriched with ABRE motifs [41] and enhanced expression of *ABI5* is prevalent during seed maturation [42]. Interestingly, the ACGTABREMOTIFA2OSEM (in both genotypes) and G-box-like (only in AC Domain) motifs are enriched in both embryo and endosperm clusters (Aem5, Aen8–10, Amn15 and Amn16; Rem12, Ren12 and Rmn15) while the prevalence of ABREZMRAB28 appeared to be endosperm specific in both genotypes (Aen2 and Amn7; Ren2 and Rmn7). Given that the seeds of AC Domain are dormant at maturity [19, 20] (Fig. 1c), our data might suggest the significance of ABA mediated transcriptional regulation specifically via G-box-like motif in the control of dormancy induction and maintenance in maturing wheat seeds of AC Domain. Although ABA has also been implicated in the control of starch synthesis during endosperm maturation [9], genes in the endospermic clusters that are enriched with ACGTABREMOTIFA2OSEM and ABREZMRAB28 motif appeared to show similar expression pattern between the two genotypes. It is therefore likely that variation in seed size/weight between the two genotypes is not influenced by ABA induced transcriptional programs mediated by the two motifs. The AC Domain gene clusters enriched with ACGTABREMOTIFA2OSEM (*P* = 10e^−3^ ~ 10e^−9^) and G-box-like (*P* = 10e^−3^ ~ 10e^−5^) motifs exhibited peak expression at the mid and late phases in the embryo and endosperm, respectively (Fig. 4). Similar expression patterns were also observed for ACGTABREMOTIFA2OSEM enriched (*P* = 10e^−3^ ~ 10e^−4^) embryo and endosperm clusters of RL4452, except that the embryo cluster (Rem12) exhibited enhanced expression through the late phase of seed maturation (Fig. 5). Given that some of these clusters are enriched with translation GO terms, our data might suggest that temporally distinct ABA regulated post-transcriptional mechanisms characterize tissue types. Furthermore, the difference between the two genotypes in the temporal window of the ABA mediated post-transcriptional regulation of embryonic genes might contribute to their variation in seed dormancy. However, the role of ABA in the control of the translation machinery during wheat seed maturation needs to be elucidated. The endospermic clusters characterized by overrepresentation of the ABREZMRAB28 motif (*P* = 10e^−3^ ~ 10e^−4^) are enriched with photosynthesis related GO terms and exhibited peak expression specifically at the early phase of seed maturation in both genotypes (Figs. 4 and 5), implying the role of ABA in modulating photosynthetic activity in the pericarp [32], a constituent of the endosperm in this study. Although the pericarp is suggested to play a key role in photosynthesis and thereby providing storage reserves to the developing endosperm [43], it appears from their expression pattern that the pericarp localized photosynthetic genes do not contribute to the difference in seed size/weight between the two genotypes.

#### Genotypically distinct motifs suggest gene regulatory networks underlying variation in seed desiccation tolerance and dormancy

Our analysis also revealed genotype specific enrichment of promoter motifs in gene clusters. For example, the DRECRTCOREAT motif (RCCGAC), which acts as a binding site for DREB/CBF TFs [44], is enriched (*P* = 10e^−3^) specifically in the endosperm clusters of RL4452 (Ren3 and Rmn8) (Figs. 8 and 9) that showed peak expression at the early phase and the expression remain elevated through the mid phase of seed maturation (Fig. 5). The HSE-perfect-type motif (NTTCNNGAANNTTCN), which acts as a binding site for members of heat shock factors (HSF) such as HsfA1a [45], is enriched (*P* = 10e^−4^ ~ 10e^−5^) specifically in the embryo and endosperm clusters of RL4452 (Rem1, Rem2, Ren2 and Rmn13) that exhibit peak expression at the early phase of seed maturation. Given that the DREB and HSF TFs are co-induced by dehydration and desiccation [44, 46, 47] and the rate of seed desiccation is more pronounced in RL4452 than AC Domain (Fig. 1), the enrichment of DRECRTCOREAT and HSF-perfect-type motifs specifically in RL4452 might suggest the role of DREB/CBF and HSF in the acquisition of desiccation tolerance. The GA response element (GARE2, RTAACARANTCYGG) motif, which contains the core TAACAAA sequence and acts as a binding site for GAMYB in the promoters of GA regulated *α-amylase* genes [48, 49], is overrepresented (*P* = 10e^−3^) specifically in the endosperm clusters of RL4452 (Ren8 and Rmn9) that show peak expression at the mid phase, and this might form a part of the transcriptional mechanisms underlying the difference in seed response to GA, which has been implicated in playing a critical role in the regulation of seed dormancy [50].

#### bZIP TFs mediating ABA regulated embryonic but not endospermic gene networks of maturing seeds appear to vary with genotypic seed dormancy status

**Fig. 10:**
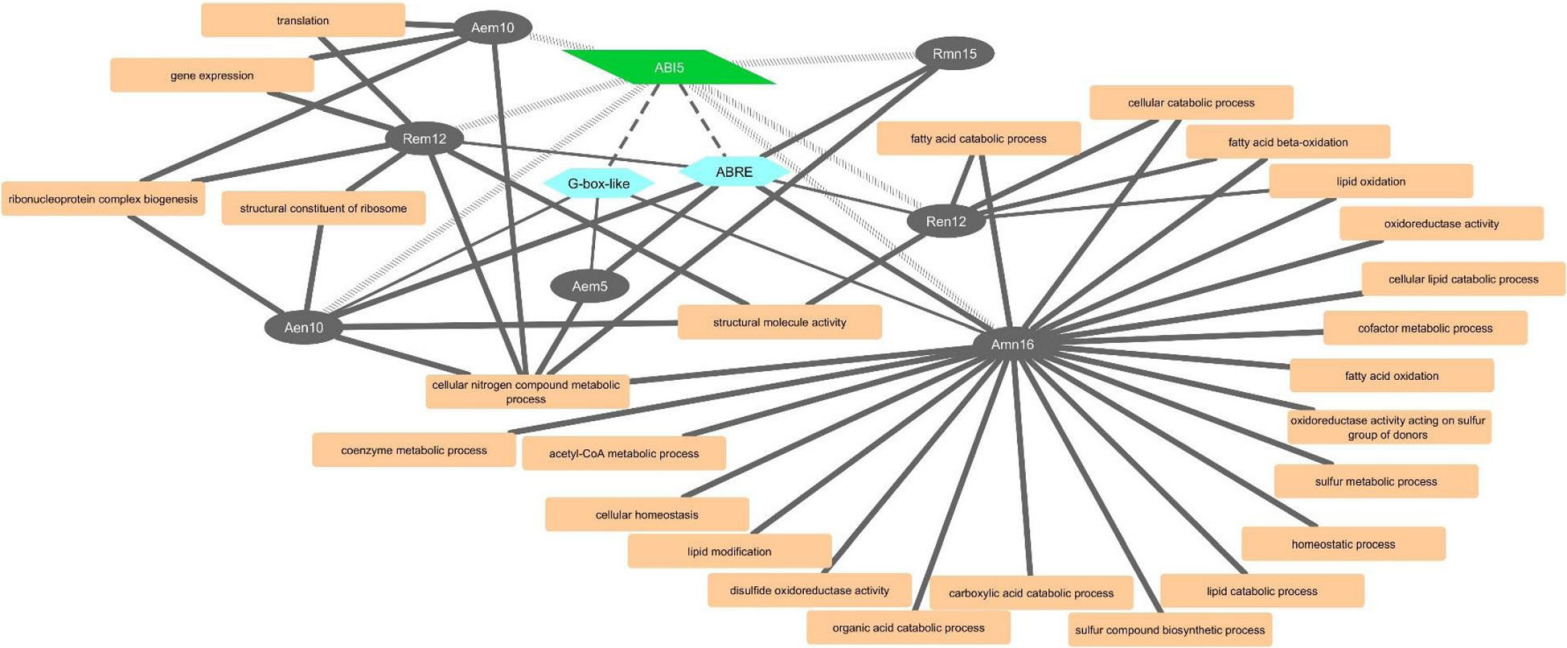
Predicted ABI5-ABRE mediated gene regulatory network during seed maturation. The transcription factor ABI5 is represented by the green parallelogram, the ABRE motif by the light blue hexagon, the seed maturation gene clusters by grey oval, and GO terms by the orange rectangles. Solid lines connect GO terms and motifs enriched in the respective gene cluster (*P* < 10e^−3^); the thickness of solid lines represent the *P* value in which thicker lines correspond to lower *P* values. Slash lines represent co-expression of ABI5 with the corresponding gene cluster. Dashed lines connect the ABI5 transcription factor with the predicted binding motifs

To identify TFs targeting the motifs identified, we referred to the PlantTFDB [51], which consists of amino acid sequences of 1940 wheat TFs belonging to 56 families. Blast searching of these TFs against PLEXdb revealed the presence of 285 TF probesets on Wheat GeneChip (E-value <1e^−100^) encoding 49 TF families. It appeared from our data that 208 and 210 TF probesets are expressed in the embryo and endosperm tissues of AC Domain, respectively, at one or more time points during seed maturation; while 216 and 210 TF probesets are expressed in the embryo and endosperm tissues of RL4452, respectively. Of these probesets, 206 and 201 are found to be expressed in the embryo and endosperm of both genotypes, respectively (Additional file 10: Table S4). Our cluster analysis showed that the *ABI5* probeset (Ta.23671.1.S1_x_at) is co-expressed with probesets in ABRE enriched embryonic and endospermic clusters (Aen10, Rem12 and Ren12), which are also enriched with several GO terms (Fig. 10, Table 1), suggesting the significance of ABI5*-* and ABRE-mediated gene network in regulating maturation associated molecular functions. However, ABI5 is absent in the ABRE enriched embryonic cluster of AC Domain (Aem5) (Table 1). Given that seeds of AC Domain are dormant at maturity (Fig. 1c) and the induction of this adaptive trait during seed maturation is regulated by ABA [52], our data might imply that ABA regulation of dormancy during seed maturation in this genotype is mediated by distinct transcriptional mechanisms. Consistent with this hypothesis, another bZIP TF, *G-box binding factor1* (*GBF1*), is co-expressed with probesets in the ABRE enriched embryonic clusters of AC Domain (Aem5). Alternatively, the absence of *ABI5* in the ABRE enriched Aem5 cluster might imply its genotype-specific post-transcriptional regulation, for example, through phosphorylation or protein degradation [53–55]. The *ABI5* probeset is also co-expressed with probesets in the embryonic clusters of AC Domain (Aem10) and RL4452 (Rem10), which exhibited peak expression at the late phase of seed maturation and are enriched with translation GO terms (Fig. 10). Although the ABRE motif is not overrepresented in these clusters, our result might imply the role of ABA in mediating post-transcriptional control of molecular functions during seed desiccation irrespective of genotype.

**Table 1:** bZIP transcription factor probesets and the respective co-expressed gene clusters of maturing seeds in AC Domain and RL4452^a^

Probeset ID	AC Domain			RL4452			Arabidopsis		
	Aem	Aen	Amn	Rem	Ren	Rmn	AGI ID	E-Value	Gene name
Ta.808.1.S1_at	Aem16	Aen14	Amn4	Rem13	Ren9	Rmn1	AT5G06950.4	1E-104	AHBP-1B
Ta.161.1.S1_at	Aem1	Aen4	Amn12	Rem1	Ren2	Rmn13	AT2G35530.1	6E-72	bZIP transcription factor family protein
Ta.10054.1.S1_at	Aem10	***Aen9***	Amn10	***Rem12***	Ren15	Rmn10	AT2G36270.1	8E-55	ABI5 (ABA INSENSITIVE 5)
Ta.23671.1.S1_x_at	Aem10	***Aen10***	***Amn16***	***Rem12***	***Ren12***	***Rmn15***	AT2G36270.1	1E-44	ABI5 (ABA INSENSITIVE 5)
Ta.29331.1.S1_a_at	Aem1	Aen2	Amn12	Rem1	Ren2	Rmn8	AT3G56850.1	8E-29	AREB3 (ABA-RESPONSIVE ELEMENT BINDING PROTEIN 3)
Ta.29331.1.S1_at	Aem1	Aen2	Amn1	Rem1	Ren16	Rmn1	AT3G56850.1	8E-29	AREB3 (ABA-RESPONSIVE ELEMENT BINDING PROTEIN 3)
Ta.5410.1.S1_at	Aem15	***Aen8***	Amn10	***Rem12***	Ren15	Rmn10	AT5G06950.4	1E-135	AHBP-1B
Ta.1612.1.S1_at	Aem1	Aen14	Amn1	Rem7	Ren7	Rmn3	AT2G35530.1	2E-68	bZIP transcription factor family protein
Ta.23670.1.A1_at	NA^b^	NA	NA	NA	NA	NA	AT5G06950.4	1E-133	AHBP-1B
Ta.4604.1.S1_at	Aem1	Aen13	Amn1	Rem1	Ren6	Rmn1	AT1G06850.1	3E-47	AtbZIP52 (*Arabidopsis thaliana* basic leucine zipper 52)
Ta.6518.1.S1_at	Aem1	Aen14	Amn6	Rem1	Ren17	Rmn7	AT4G38900.3	2E-80	bZIP protein
TaAffx.24695.1.S1_at	Aem15	Aen2	Amn7	Rem6	Ren3	Rmn8	AT5G24800.1	5E-35	BZIP9 (BASIC LEUCINE ZIPPER 9)
TaAffx.88821.1.S1_at	Aem15	***Aen9***	Amn13	Rem13	Ren13	Rmn11	AT5G06950.4	1E-101	AHBP-1B
Ta.100.1.S1_at	Aem10	***Aen10***	Amn5	Rem9	***Ren12***	Rmn4	AT4G36730.1	1E-35	GBF1
Ta.13267.1.S1_at	NA	NA	NA	Rem10	NA	Rmn5	AT4G35040.1	1E-43	bZIP transcription factor family protein
Ta.13357.2.S1_at	Aem6	Aen1	Amn2	Rem5	Ren16	Rmn2	AT2G46270.1	6E-48	GBF3 (G-BOX BINDING FACTOR 3)
Ta.140.1.S1_at	***Aem5***	Aen11	***Amn16***	Rem5	Ren7	Rmn14	AT4G36730.1	3E-27	GBF1
Ta.19597.1.S1_at	Aem14	***Aen8***	***Amn16***	***Rem12***	Ren9	Rmn10	AT1G45249.1	1E-35	ABF2 (ABSCISIC ACID RESPONSIVE ELEMENTS-BINDING FACTOR 2)
Ta.19597.1.S1_x_at	Aem14	***Aen10***	Amn10	Rem14	Ren18	Rmn12	AT1G45249.1	1E-35	ABF2 (ABSCISIC ACID RESPONSIVE ELEMENTS-BINDING FACTOR 2)
Ta.23670.1.S1_at	Aem1	NA	Amn6	Rem1	NA	Rmn1	AT5G06950.4	1E-133	AHBP-1B
Ta.25454.1.S1_at	Aem1	NA	Amn1	Rem2	NA	Rmn2	AT2G42380.2	1E-49	bZIP transcription factor family protein
Ta.28910.1.S1_s_at	Aem3	Aen4	Amn2	Rem7	Ren6	Rmn3	AT2G35530.1	2E-64	bZIP transcription factor family protein
Ta.6443.2.S1_at	Aem8	Aen12	Amn3	Rem7	Ren13	***Rmn15***	AT2G40950.1	7E-62	BZIP17
Ta.6919.1.S1_at	Aem11	***Aen9***	Amn13	Rem9	Ren13	Rmn4	AT1G58110.2	6E-60	bZIP family transcription factor
Ta.893.1.S1_at	NA	Aen2	Amn8	NA	Ren3	Rmn8	AT5G28770.1	1E-30	BZO2H3

^a^The amino acid sequences of the wheat transcription factors available in the Plant Transcription Factor database (PlantTFDB 3.0) were searched against the wheat microarray platform in the Plant Expression Database (PLEXdb) to identify the respective probeset (<e^−100^)

^b^ *NA* not expressed in either or both tissues as determined by MAS5.0. Bold italicized clusters represent clusters enriched with ABRE motifs

#### SITEIIATCYTC motif suggests genotypically overlapping role for TCP TFs in post-transcriptional regulation of embryo maturation

**Fig. 11:**
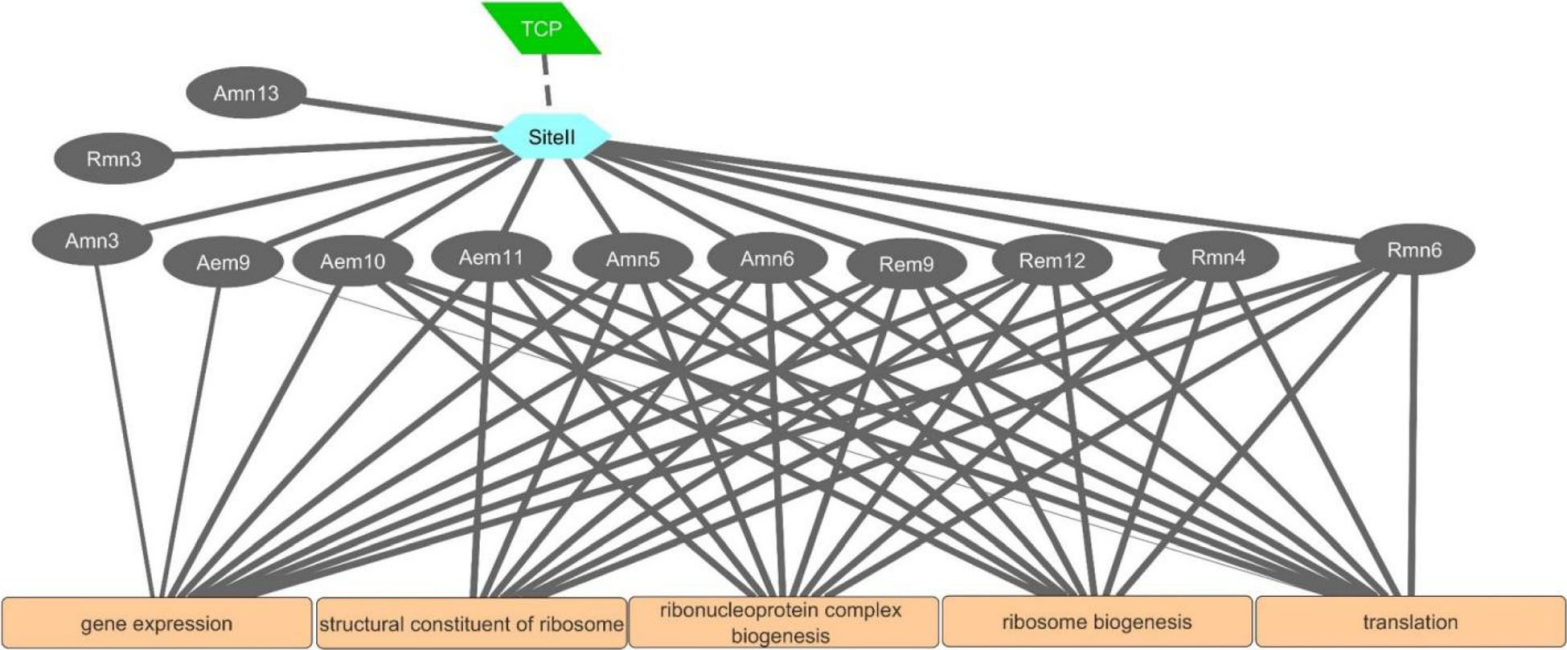
Predicted TCP-Site II mediated gene regulatory network during seed maturation. The transcription factor TCP is represented by the green parallelogram, the Site II motif by the light blue hexagon, the seed maturation gene clusters by grey ovals and GO terms by the orange rectangles. Solid lines connect GO terms and motifs enriched in the respective gene cluster (*P* < 10e^−3^); the thickness of solid lines represent the *P* value in which thicker lines correspond to lower *P* values. Dashed lines connect the TCP transcription factor with the predicted binding motifs

The SITEIIATCYTC motif (TGGGCY), which acts as a target for TEOSINTE BRANCHED1 (TB1), CYCLOIDEA (CYC) and PROLIFERATING CELL NUCLEAR ANTIGEN FACTOR1 (PCF) (TCP) TFs [56], is enriched (*P* = 10e^−3^ ~ 10e^−11^) in several embryo and endosperm clusters of both genotypes that showed peak expression especially during the mid to late phases of seed maturation (Figs. 8 and 9). Interestingly, this motif is not enriched in Amn7–11 and Rmn7–12 clusters that consist of probesets predominantly expressed in the endosperm with peak expressions occurring at different time points (Figs. 4 and 5), suggesting that genes with SITEIIATCYTC motif are embryo specific in maturing wheat seeds, however, further study is required to clarify this hypothesis. Using the amino acid sequences of 14 wheat TCPs available in the PlantTFDB, we were unable to identify a *TCP* probeset on Wheat GeneChip. However, gene clusters enriched with the SITEIIATCYTC motif are also found to be enriched with ribosome biogenesis and structure, gene expression and translation GO terms (Fig. 11), suggesting the role of TCP in the post-transcriptional regulation of embryonic molecular functions during the mid to late phases of seed maturation irrespective of the genotypic variation in dormancy and seed size/weight, likely to facilitate the synthesis of proteins required for acquisition of desiccation tolerance. Consistently, the SITEIIATCYTC motif is found in genes related to protein synthesis [56, 57]. Furthermore, the UP1 site (GGCCCAWW), which is almost identical with the SITEIIATCYTC motif [56, 58], has been shown to be overrepresented in clusters consisting of protein synthesis related genes upregulated in germinating seeds, and the SITEIIATCYTC targeting TCP14 regulates embryo growth potential during germination [59].

#### Genotypic distribution of gene expression reveals distinct and overlapping transcriptional programs and regulatory networks underlying variation in seed dormancy and size/weight

To gain further insights into distinct and overlapping transcriptional programs and regulatory networks underlying seed maturation between the two genotypes, we determined the distribution of probesets in each of the AC Domain cluster across the embryo and endosperm clusters of RL4452 and vice versa (Additional files 3, 4, 5 and 6: Figs. S3–S6). Our result shows that over 50% of the probesets in AC Domain embryo clusters exhibiting peak expression at early (Aem1) and mid to late (Aem10 and Aem11) phases of seed maturation are overrepresented in the RL4452 embryo clusters with peak expression at early (Rem1) and mid to late (Rem9, Rem10 and Rem12) phases (Additional file 3: Figs. S3). Probesets overlapping between the Aem1 and Rem1 clusters are enriched with chromatin/chromosome (*P* = 4.2e^−28^ ~ 2.9e^−24^) and protein-DNA complex assembly (*P* = 1.6e^−29^) GO terms while those overlapping between Aem10 and Aem11, and Rem9, Rem10 and Rem12 clusters are enriched with ribosome biogenesis and translation (*P* = 2e^−14^ ~ 8.9e^−14^) GO terms (Additional file 11: Table S5), indicating that gene regulatory networks underlying embryo growth during the early phase and post-transcriptional regulation of molecular functions during the late phase of seed maturation are temporally conserved between the two genotypes despite their difference in seed dormancy and size/weight. Consistently, proteomic analysis of developing embryos of rice indicated that proteins involved in cell growth/division are highly expressed during the early stages of rice embryo development [60]. Furthermore, enrichment of chromatin assembly GO term or upregulation of the related genes has been reported in germinating wheat and barley seeds that are characterized by growing/expanding embryos [34]. In contrast, embryonic clusters of AC Domain with peak expression at mid-phase (30 DAA) of seed maturation (Aem4 and Aem5) are distributed not only to the corresponding clusters of RL4452 (Rem4 and Rem5) that show similar expression pattern, but also to the adjacent clusters (Rem3 and Rem6); for example, over 21% of probesets in Aem5 cluster are overrepresented in Rem3 cluster that show peak expression mainly at the early phase, suggesting that temporally distinct transcriptional reprograming in maturing embryos characterizes genotypic differences in seed dormancy. Furthermore, although the Aem5 cluster is enriched with ACGTABREMOTIFA2OSEM motif (Fig. 8), only ~1% of its probesets is found in the ACGTABREMOTIFA2OSEM enriched embryo cluster of RL4452 (Rem12) that exhibited peak expression during the mid to late (30 to 50 DAA) phases of seed maturation (Fig. 9). Likewise, over 50% of probesets in Rem12 cluster are overrepresented in the Aem9 and Aem10 clusters, which show peak expression during the mid to late (40 to 50 DAA) phases (Fig. 4), while only <1% Rem12 probesets are distributed to Aem5 cluster (Additional files 5 and 6: Figs. S5 and S6). Since ABA is a major player for the establishment of dormancy during seed maturation [52], these results might imply that temporally distinct, yet overlapping, ABA regulated embryonic gene networks in maturing seeds underlie the variation in the induction and maintenance of dormancy between the two genotypes.

Similar to that observed in the embryo, over 40% of the probesets in AC Domain endospermic clusters with peak expression in the early (Aen1 and Aen2) and late (Aen9 and Aen10) phases of seed maturation (Fig. 4) are overrepresented in the RL4452 clusters that exhibit peak expression at the early (Ren1, Ren2 and Ren3) and late (Ren12 and 13) phases, respectively (Additional file 4: Figs. S4). Probesets overlapping between the endospermic clusters of both genotypes exhibiting peak expression at the early phase are enriched with photosynthesis (*P* = 1.1e^−21^) GO term (Additional file 12: Table S6), suggesting that pericarp derived photosynthate does not contribute to the difference in seed size/weight between the two genotypes. On the other hand, probesets overrepresented in the AC Domain endospermic clusters exhibiting peak expression at the mid phase (Aen4 and Aen5) are distributed not only to the corresponding RL4452 clusters (Ren4, Ren5 and Ren6) but also to clusters that show peak expression primarily at early phase of seed maturation (Ren2 and Ren3). Given that probesets overlapping between all these clusters are enriched with nutrient reservoir activity (GO:0045735; *P* = 1.8e^−15^) GO term (Additional file 12: Table S6), our results suggest that transcriptional programs underlying reactions or pathways involved in the storage of nutritious substrates are shared by the two genotypes but operate in a temporally distinct manner that may affect seed size/weight. Unlike that observed in the embryo, over 30% of the probesets in each of the ABRE enriched endospermic clusters of AC Domain with peak expression at the late phase (Aen9 and Aen10) are shared with the ABRE enriched endospermic cluster of RL4452 (Ren12) exhibiting peak expression at the same phase of seed maturation. Since probesets overlapping between the Aen9, Aen10 and Ren12 clusters are enriched with gene expression and translation (*P* = 3.1e^−10^ ~ 8.3e^−8^) GO terms (Additional file 12: Table S6), our data suggest that ABA regulated endospermic gene networks underlying post-transcriptional regulation of molecular functions during the late phase of seed maturation are conserved both genotypically and temporally, and therefore exert minimal effect on seed dormancy and size/weight.

## Conclusions

The present study showed that temporal shifts in gene expression within the embryo and endosperm tissues vary with genotype, implying their role in governing phenotypic variations in seed size/weight and dormancy. However, tissue types appeared to be characterized by distinct but temporally and genotypically overlapping expression profiles and therefore molecular functions. It can be inferred from our data that genotypically distinct ABA and GA regulated gene networks modulate starch biosynthesis and acquisition of dormancy, leading to variations in seed weight/size and tolerance to preharvest sprouting, respectively. Our ABRE motif and bZIP TF data imply that genotypically distinct ABA regulated gene networks underlie the induction of dormancy during wheat seed maturation. Given that maturation associated biological processes significantly affect seed yield and quality, the findings of this study advance our knowledge of the transcriptional programs and regulatory networks regulating seed dormancy and seed size/weight during the maturation phase of seed development in wheat, a critical step to design molecular strategies for improving its yield and quality.

## Methods

### Plant material and growth conditions

Wheat plants of two genotypes with spring growth habit, AC Domain (a cultivar registered and widely cultivated in western Canada) and RL4452 (unregistered backcross derivative of ‘Glenlea’ and ‘Kitt’ cultivars [Glenlea*6/Kitt]) [61], were grown and managed as described previously^18^ except that a growth room under a 22 °C/18 °C (day/night) with a 16/8 h photoperiod was used. Seeds of the two genotypes were obtained from Dr. Mark Jordan of Agriculture and Agri-Food Canada (AAFC)-Morden Research and Development Center (Morden, Manitoba, Canada). Seed developmental stages were determined using the first extrusion of the yellow anthers in the spikes as 0 DAA; maturing seeds were then harvested at 20, 30, 40 and 50 DAA. The endosperm (including the aleurone and pericarp) and the embryo (including the scutellum) were dissected from maturing seeds harvested from the middle region of each spike in liquid nitrogen, and then stored at -80 °C until RNA extraction. Two to three independent biological replicates were collected from each tissue and genotype at each time point of seed maturation (~40 seeds per 2–3 spikes per 2–3 plants per replicate for 20, 30 and 40 DAA samples; ~100–120 seeds per 4–6 spikes per 4–6 plants per replicate for 50 DAA samples). Seed germination assays were performed as described previously [62].

### Changes in seed weights and moisture content

Changes in fresh and dry weights of maturing seeds of the two genotypes were determined at each time point. Fresh weights were obtained from 20 to 23 seeds by weighing the individual maturing seed on a four decimal place analytical balance (Denver Instrument, Bohemia, NY, USA). To determine dry weights, the same seed samples were dried in an oven at 105 °C for 72 h and then reweighed. Moisture content was determined as a percentage of seed fresh weight. Test for statistically significant differences in seed fresh weight, dry weight and moisture content between the two genotypes was performed using t-Student test at a probability of *P* < 0.05.

### RNA extraction

Total RNA samples of the embryonic tissues were extracted using the RNeasy Plant Mini Kit (Qiagen, Hilden, Germany). The integrity and purity of the resulting total RNA samples was verified by gel electrophoresis and spectrophotometrically before the samples are used for the microarray analysis. For the endosperm, total RNA samples were first extracted as described previously [17, 63]. The RNA samples were digested with DNase (Ambion, Austin, TX, USA) at 37 °C for 30 min to eliminate genomic DNA contamination. The purity and concentration, and integrity of the endospermic RNA samples was determined as described above. The RNA samples treated with DNase were then subjected to mRNA isolation, which was performed using PolyATract Kit (Promega, Madison, WI, USA) following the manufacturer’s protocol.

### DNA microarray analysis

The total RNA and mRNA samples of the embryo and the endosperm, respectively, were subjected to labeling and hybridization to the Affymetrix GeneChip Wheat Genome Array (Affymetrix, Santa Clara, CA, USA) exactly as described previously [64]. After hybridization, washing and staining of the arrays was performed in an Affymetrix Fluidics Station 450 following the manufacturer’s protocol. Arrays were then scanned with an Affymetrix Scanner 3000. The microarray experiment for each tissue derived from each genotype at each stage of seed maturation involved at least two independent biological replicates.

#### Data analysis

Converting the data from all probe pairs into as single hybridization intensity and representation in CEL format, and adjustment of the total signal intensity per chip and determination of the number of probesets with present detection call was performed using the Affymetrix GeneChip Operating Software as described before [64]. Reproducibility of the data from the independent biological replicates was confirmed with scatter plot expression analysis. The MAS5 statistical algorithm of FlexArray was used to determine the number of probesets expressed in each tissue of both genotypes at least at one stage of seed maturation in all replications (*P* < 0.05). Following normalization of the raw data with the Robust Multi-array Average (RMA) procedure, the average RMA value of each probeset was used to perform Principal Component Analysis using FlexArray, and hierarchical clustering using MultiExperiment Viewer (MeV) software version 4.9 [65] with default setting. The FlexArray software version 1.6.3 [66] was used to calculate the expression level of each probeset at different time points of seed maturation relative to that exhibiting the highest RMA normalized signal intensity, which was arbitrarily set to a value of 1. The relative expression values were subjected to cluster analysis by K-means clustering and Pearson correlation coefficient using MeV software; MeV was also used to generate heat maps.

#### Gene ontology and motif enrichment analysis

GO enrichment for each gene cluster was performed with the AgriGO analysis toolkit (http://bioinfo.cau.edu.cn/agriGO/analysis.php) [67] using the default parameters of Fisher exact test (P < 0.05) and False Discovery Rate (FDR) correction by Yekutieli method. Candidate gene annotations for each probeset was obtained using HarvEST Wheat Chip version 1.59 (http://harvest.ucr.edu/) [68]. Promoter motif enrichment for each gene cluster was performed with Osiris [38] using the 500 bp upstream promoter region of the corresponding rice homologs in each cluster (*P* < 10e^−30^). Visualization of the predictive gene regulatory networks was generated using Cytoscape (version 3.2.1) [69].

#### Identification of transcription factor probesets

To identify probesets representing TFs on Wheat GeneChip, amino acid sequences of wheat TFs available in the Plant TF Database version 3.0 (PlantTFDB; http://planttfdb.cbi.pku.edu.cn/index.php?sp=Tae) [51] were blast searched against the Plant Expression Database (PLEXdb; http://www.plexdb.org/index.php) [70] using E-value <1e^−100^.

### Validation of microarray results by qPCR

Validation of the microarray data was performed with 10 randomly selected differentially expressed probesets using real-time qPCR and the same RNA samples used for the microarray analysis. Preparation of the cDNA samples and the qPCR assay was performed as described previously [71] using thermal cycling conditions described before [19]. Relative transcript level of the target genes was determined as described in Livak and Schmittgen [72] using *β-actin* as a reference gene. DNA sequences of the specific probeset IDs were obtained from HarvEST and used for designing the qPCR primers (Additional file 13: Table S7).

## Additional files

Additional file 1: **Fig. S1.** Validation of the microarray data with qPCR. (PDF 116 kb)

Additional file 2: **Fig. S2.** Hierarchical clustering of the embryo and endosperm by expression patterns of probesets during seed maturation. (PDF 76 kb)

Additional file 3: **Fig. S3.** Distribution of probesets in each AC Domain embryonic cluster across the RL4452 clusters. (PDF 271 kb)

Additional file 4: **Fig. S4.** Distribution of probesets in each AC Domain endospermic cluster across the RL4452 clusters. (PDF 298 kb)

Additional file 5: **Fig. S5.** Distribution of probesets in each RL4452 embryonic cluster across the AC Domain clusters. (PDF 369 kb)

Additional file 6: **Fig. S6.** Distribution of probesets in each RL4452 endospermic cluster across the AC Domain clusters. (PDF 416 kb)

Additional file 7: **Table S1.** Robust multi-array average values and gene clusters for probesets expressed in the embryo and endosperm of maturing AC Domain and RL4452 seeds. (XLS 24009 kb)

Additional file 8: **Table S2.** Gene ontology enrichment in the embryo and/or endosperm clusters of AC Domain and RL4452. (XLS 279 kb)

Additional file 9: **Table S3.** Promoter motif enrichment in the embryo and endosperm clusters of AC Domain and RL4452. (XLS 46 kb)

Additional file 10: **Table S4.** Identification and annotation of wheat transcription factor probesets. (XLS 300 kb)

Additional file 11: **Table S5.** Gene ontology enrichment of probesets overlapping between embryo clusters of AC Domain and RL4452. (XLS 2627 kb)

Additional file 12: **Table S6.** Gene ontology enrichment of probesets overlapping between endosperm clusters of AC Domain and RL4452. (XLS 4007 kb)

Additional file 13: **Table S7.** qPCR primers used for validation of the microarray data. (XLS 37 kb)

## Abbreviations

ABA: Abscisic acid
ABRE: ABA responsive element
DAA: Days after anthesis
GA: Gibberellin
GO: Gene ontology
PlantTFDB: Plant Transcription Factor database
RMA: Robust Multi-array Average
TF: Transcription factor
