# Supplementary material for: Seed maturation associated transcriptional programs and regulatory networks underlying genotypic difference in seed dormancy and size/weight in wheat (Triticum aestivum L.)
Source: BMC Plant Biol. 2017 Sep 16;17:154. doi: 10.1186/s12870-017-1104-5 (PMC5603048; doi:10.1186/s12870-017-1104-5)
Supplement: Supplementary file 1 — Fig. S1. Validation of the microarray data with qPCR. (PDF 116 kb) [file 12870_2017_1104_MOESM1_ESM.pdf]

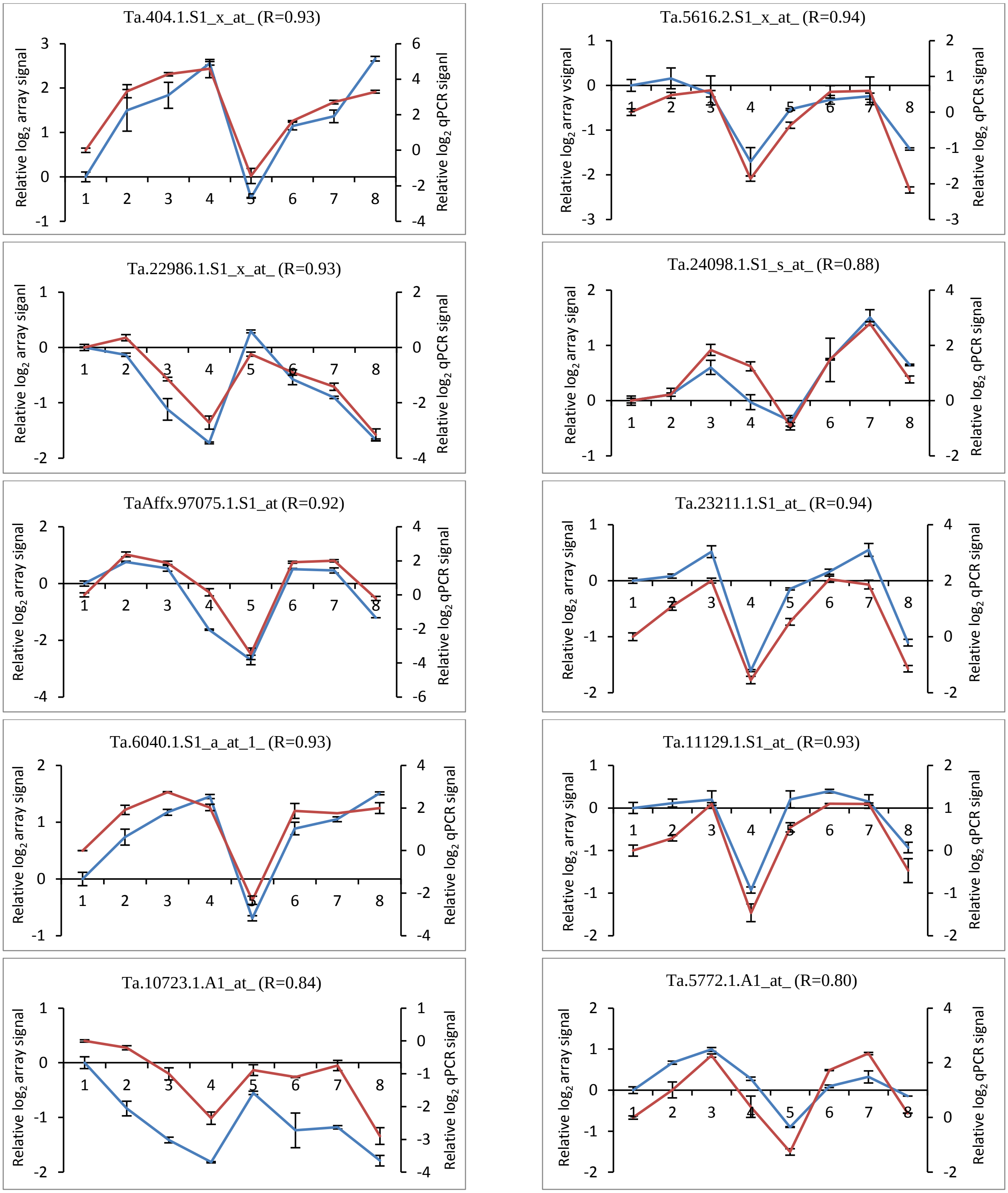

Figure S1. Validation of the microarray data with qPCR. The qPCR analysis was performed for selected probesets in the embryo samples of both geneotypes and the resulting data were compared with the corresponding microarray data. Blue lines represents microarray data (left y-

axis) and red lines represents qPCR data (right y-axis) for both AC Domain and RL4452 embryos at different seed maturation time points. Siganl intensities for each probest in both microarray and qPCR analysis were expressed relative to that of AC Domain embryo at 20 DAA, which was arbitrarily set to a value of 0. Data are means of 3 biological replicates (two technical replicate for each biological replicate) ± SE. The probeset IDs and the pearson correlation coeffcient (R) between the microarray and qPCR data are indicated in each graph. Annotation of the probesets used for the validation experiment can be found in Table S7. The numbers on the x-axis represent the embryo samples at different seed maturation time points: 1, AC Domain 20 DAA; 2, AC Domain 30 DAA; 3, AC Domain 40 DAA; 4, AC Domain 50 DAA; 5, RL4452 20 DAA; 6, RL4452 30 DAA; 7, RL4452 40 DAA; and 8, RL4452 50 DAA.
