# Supplementary material for: Seed maturation associated transcriptional programs and regulatory networks underlying genotypic difference in seed dormancy and size/weight in wheat (Triticum aestivum L.)
Source: BMC Plant Biol. 2017 Sep 16;17:154. doi: 10.1186/s12870-017-1104-5 (PMC5603048; doi:10.1186/s12870-017-1104-5)
Supplement: Supplementary file 2 — Fig. S2. Hierarchical clustering of the embryo and endosperm by expression patterns of probesets during seed maturation. (PDF 76 kb) [file 12870_2017_1104_MOESM2_ESM.pdf]

a) AC Domain

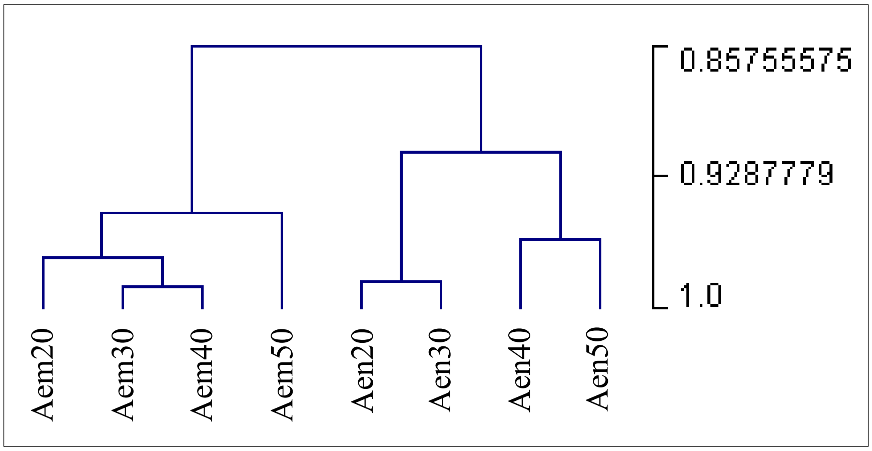

b) RL4452

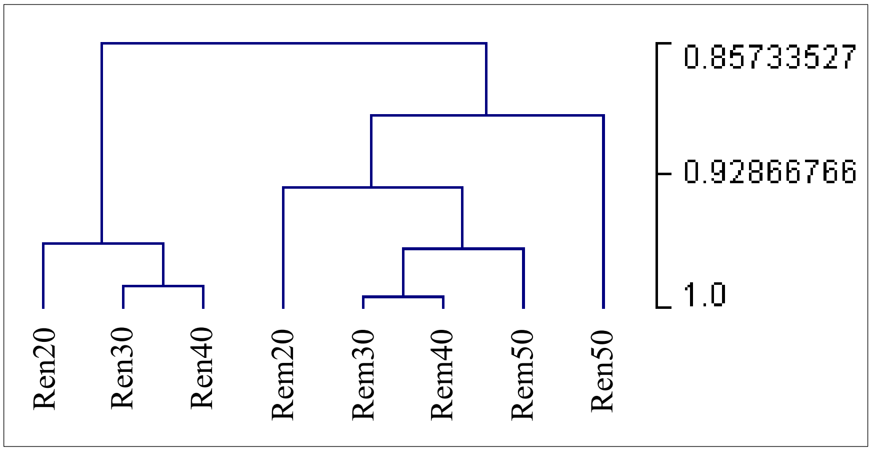

Figure S2. Hierarchical clustering of the embryo and endosperm by expression patterns of probesets during seed maturation. Hierarchical tree of clusters showing relationship between maturing embryo and endosperm of AC Domain (A) and RL4452 (B). The scale shows node height for a particular inter-node distance. Aem, AC Domain embryo; Aen, AC Domain endosperm; Rem, RL4452 embryo; Ren, RL4452 endosperm at 20, 30, 40 and 50 days after anthesis (DAA).
