# Supplementary material for: Seed maturation associated transcriptional programs and regulatory networks underlying genotypic difference in seed dormancy and size/weight in wheat (Triticum aestivum L.)
Source: BMC Plant Biol. 2017 Sep 16;17:154. doi: 10.1186/s12870-017-1104-5 (PMC5603048; doi:10.1186/s12870-017-1104-5)
Supplement: Supplementary file 3 — Fig. S3. Distribution of probesets in each AC Domain embryonic cluster across the RL4452 clusters. (PDF 271 kb) [file 12870_2017_1104_MOESM3_ESM.pdf]

AC Domain Embryo		RL4452 Embryo: 20				30			40	50		Constitutive Multiple				
		Rem1	Rem2	Rem3	Rem4	Rem5	Rem6	Rem7	Rem8	Rem9	Rem10	Rem11	Rem12	Rem13	Rem14	N/A
20	Aem1	56.31%	24.24%	2.58%	2.55%	0.89%	0.29%	0.51%	0.48%	0.41%	1.46%	2.01%	0.45%	3.15%	0.41%	4.27%
	Aem2	13.40%	33.82%	17.46%	8.97%	8.73%	2.40%	2.80%	0.65%	0.45%	0.57%	4.22%	0.57%	1.71%	1.02%	3.25%
	Aem3	6.44%	23.70%	28.79%	3.16%	6.90%	1.52%	4.86%	2.28%	0.70%	1.11%	15.04%	0.70%	1.23%	0.82%	2.75%
30	Aem4	9.76%	9.84%	5.13%	11.61%	14.55%	13.96%	4.79%	3.03%	2.10%	2.44%	2.78%	2.69%	3.03%	4.46%	9.84%
	Aem5	2.70%	7.97%	21.28%	4.02%	20.55%	8.96%	13.70%	2.96%	0.59%	0.40%	10.67%	1.32%	0.66%	0.99%	3.23%
40	Aem6	0.93%	2.43%	2.72%	3.43%	11.95%	23.82%	14.95%	14.31%	1.57%	1.14%	4.58%	7.94%	0.86%	3.58%	5.79%
	Aem7	1.57%	2.99%	10.21%	1.41%	11.37%	5.71%	30.64%	7.86%	0.94%	0.52%	20.74%	1.41%	0.31%	0.79%	3.51%
	Aem8	1.20%	1.20%	1.85%	1.30%	4.34%	8.78%	28.28%	29.88%	2.39%	0.80%	6.38%	6.78%	0.75%	1.35%	4.74%
	Aem9	1.03%	0.92%	0.71%	1.25%	2.82%	10.65%	3.31%	21.56%	9.61%	2.44%	1.47%	32.21%	1.25%	3.86%	6.90%
50	Aem10	1.18%	0.81%	0.38%	1.07%	1.18%	3.28%	0.97%	5.27%	31.27%	8.60%	0.59%	29.61%	2.42%	5.37%	8.01%
	Aem11	0.94%	0.36%	0.25%	0.43%	0.65%	2.21%	0.18%	1.70%	44.25%	21.62%	0.36%	9.58%	2.68%	7.48%	7.30%
	Aem12	2.21%	0.30%	0.23%	1.07%	0.34%	1.14%	0.17%	0.50%	13.68%	53.76%	0.17%	2.48%	8.95%	5.63%	9.36%
Constitutive Multiple	Aem13	42.89%	7.44%	1.07%	5.70%	1.24%	0.74%	0.41%	0.41%	2.31%	7.44%	0.74%	0.74%	21.57%	2.15%	5.12%
	Aem14	10.69%	2.34%	1.02%	2.49%	0.88%	2.49%	1.46%	2.93%	10.54%	22.99%	2.49%	2.64%	18.89%	2.93%	15.23%
	Aem15	2.27%	0.10%	0.59%	2.27%	2.87%	12.45%	1.58%	5.53%	14.53%	8.60%	1.58%	19.17%	2.57%	14.82%	11.07%
	Aem16	11.80%	2.34%	0.70%	2.97%	1.25%	1.25%	0.55%	0.63%	5.39%	23.75%	0.86%	1.88%	32.27%	6.88%	7.50%

Figure S3. Distribution of probesets in each AC Domain embryonic cluster across the RL4452 clusters. The number of probesets commonly expressed in each of the AC Domain embryo cluster (Aem1-16) and the RL4452 embryo clusters (Rem1-14) is calculated as a percentage of the total number of probesets expressed in a given AC Domain embryo cluster. The gradient of the red color in the fill represents change in percentage. N/A indicates the percentage of probesets in a given AC Domain embryonic cluster with no expression in RL4452 embryo.
