# Supplementary material for: Seed maturation associated transcriptional programs and regulatory networks underlying genotypic difference in seed dormancy and size/weight in wheat (Triticum aestivum L.)
Source: BMC Plant Biol. 2017 Sep 16;17:154. doi: 10.1186/s12870-017-1104-5 (PMC5603048; doi:10.1186/s12870-017-1104-5)
Supplement: Supplementary file 4 — Fig. S4. Distribution of probesets in each AC Domain endospermic cluster across the RL4452 clusters. (PDF 298 kb) [file 12870_2017_1104_MOESM4_ESM.pdf]

AC Domain Endosperm		RL4452 Endosperm 20			30			40			50				Constitutive Multiple					
		Ren1	Ren2	Ren3	Ren4	Ren5	Ren6	Ren7	Ren8	Ren9	Ren10	Ren11	Ren12	Ren13	Ren14	Ren15	Ren16	Ren17	Ren18	N/A
20	Aen1	24.53%	19.33%	7.24%	3.84%	1.24%	3.00%	3.67%	5.54%	3.00%	0.62%	1.19%	0.40%	0.73%	5.09%	2.09%	6.95%	1.92%	2.54%	7.07%
	Aen2	5.19%	22.47%	22.91%	6.11%	0.81%	6.78%	2.73%	4.49%	1.14%	0.11%	0.29%	0.07%	0.37%	14.00%	0.52%	5.82%	0.74%	0.55%	4.90%
	Aen3	1.66%	5.64%	13.18%	4.28%	1.07%	14.61%	6.41%	12.77%	1.19%	0.24%	0.18%	0.06%	0.30%	26.01%	1.01%	5.46%	0.36%	0.36%	5.23%
30	Aen4	4.11%	13.78%	18.80%	12.59%	1.94%	9.45%	3.13%	4.32%	0.49%	0.16%	0.16%	0.16%	0.54%	14.42%	0.86%	3.57%	0.86%	0.49%	10.16%
	Aen5	5.01%	11.60%	5.35%	14.11%	4.89%	13.88%	4.78%	4.55%	1.48%	0.46%	1.25%	0.46%	1.59%	6.94%	1.59%	2.84%	2.84%	1.02%	15.36%
40	Aen6	2.02%	2.69%	2.69%	3.25%	2.69%	17.38%	21.30%	15.13%	4.93%	1.57%	0.56%	0.00%	0.11%	7.85%	2.69%	3.14%	0.78%	1.35%	9.87%
	Aen7	1.86%	0.85%	0.51%	1.69%	1.86%	3.98%	21.69%	7.80%	16.78%	2.80%	4.66%	1.86%	2.29%	1.36%	13.73%	2.88%	1.44%	2.97%	8.98%
	Aen8	1.31%	0.22%	0.22%	0.27%	1.53%	0.60%	3.28%	0.87%	9.17%	7.48%	27.40%	12.12%	5.35%	0.38%	16.21%	1.26%	1.58%	4.80%	5.95%
50	Aen9	0.46%	0.14%	0.04%	0.32%	0.57%	0.07%	0.42%	0.07%	0.81%	7.00%	5.13%	32.66%	38.03%	0.07%	1.66%	0.18%	3.36%	2.90%	6.12%
	Aen10	0.54%	0.08%	0.00%	0.11%	0.16%	0.14%	0.54%	0.11%	1.44%	7.37%	15.72%	44.02%	18.70%	0.03%	3.14%	0.11%	1.33%	3.44%	3.01%
Constitutive Multiple	Aen11	4.09%	1.42%	0.18%	1.78%	6.04%	3.20%	7.10%	2.13%	6.39%	10.30%	5.86%	4.09%	7.10%	0.89%	11.01%	1.42%	6.22%	3.20%	17.58%
	Aen12	3.64%	0.52%	0.00%	0.94%	1.14%	0.21%	1.66%	0.42%	2.08%	9.36%	8.42%	20.06%	22.66%	0.10%	4.99%	0.52%	7.38%	5.09%	10.81%
	Aen13	5.09%	3.13%	2.66%	1.74%	0.93%	9.14%	15.51%	16.09%	9.03%	1.16%	2.08%	0.58%	1.04%	7.18%	4.17%	9.95%	1.27%	2.20%	7.06%
	Aen14	10.67%	4.32%	1.02%	2.41%	2.29%	2.67%	5.08%	2.80%	4.07%	5.72%	7.62%	3.68%	8.13%	1.27%	8.26%	3.56%	9.40%	8.13%	8.89%
	Aen15	7.96%	0.75%	0.65%	0.54%	1.40%	1.18%	3.12%	1.40%	9.35%	3.98%	12.90%	8.49%	8.28%	0.65%	6.34%	2.90%	8.60%	15.27%	6.24%
	Aen16	4.41%	4.01%	1.20%	6.81%	7.21%	2.00%	1.80%	0.60%	2.20%	5.41%	3.01%	2.40%	7.62%	1.20%	4.81%	1.40%	12.63%	2.20%	29.06%

Figure S4. Distribution of probesets in each AC Domain endospermic cluster across the RL4452 clusters. The number of probesets commonly expressed in each of the AC Domain endosperm cluster (Aen1-16) and the RL4452 endosperm clusters (Rem1-18) is calculated as a percentage of the total number of probesets expressed in a given AC Domain endosperm cluster. The gradient of the red color in the fill represents change in percentage. N/A indicates the percentage of probesets in a given AC Domain endospermic cluster with no expression in RL4452 endosperm.
