# Supplementary material for: Seed maturation associated transcriptional programs and regulatory networks underlying genotypic difference in seed dormancy and size/weight in wheat (Triticum aestivum L.)
Source: BMC Plant Biol. 2017 Sep 16;17:154. doi: 10.1186/s12870-017-1104-5 (PMC5603048; doi:10.1186/s12870-017-1104-5)
Supplement: Supplementary file 5 — Fig. S5. Distribution of probesets in each RL4452 embryonic cluster across the AC Domain clusters. (PDF 369 kb) [file 12870_2017_1104_MOESM5_ESM.pdf]

		AC Domain Embryo																
		20			30			40				50		Constitutive Multiple				
RL4452 Embryo		Aem1	Aem2	Aem3	Aem4	Aem5	Aem6	Aem7	Aem8	Aem9	Aem10	Aem11	Aem12	Aem13	Aem14	Aem15	Aem16	#N/A
20	Rem1	46.43%	8.67%	2.89%	3.05%	1.08%	0.34%	0.79%	0.63%	0.50%	0.58%	0.68%	1.73%	13.63%	1.92%	0.60%	3.97%	12.53%
	Rem2	28.05%	30.70%	14.93%	4.31%	4.46%	1.25%	2.10%	0.88%	0.63%	0.55%	0.37%	0.33%	3.32%	0.59%	0.04%	1.11%	6.38%
	Rem3	4.47%	23.70%	27.12%	3.36%	17.81%	2.09%	10.75%	2.04%	0.72%	0.39%	0.39%	0.39%	0.72%	0.39%	0.33%	0.50%	4.85%
30	Rem4	8.12%	22.44%	5.48%	14.01%	6.19%	4.87%	2.74%	2.64%	2.34%	2.03%	1.22%	3.25%	7.01%	1.73%	2.34%	3.86%	9.75%
	Rem5	1.78%	13.70%	7.52%	11.03%	19.89%	10.64%	13.83%	5.54%	3.31%	1.40%	1.15%	0.64%	0.96%	0.38%	1.85%	1.02%	5.35%
	Rem6	0.55%	3.62%	1.59%	10.18%	8.34%	20.42%	6.68%	10.79%	12.02%	3.74%	3.74%	2.08%	0.55%	1.04%	7.73%	0.98%	5.95%
40	Rem7	0.79%	3.39%	4.08%	2.80%	10.23%	10.28%	28.76%	27.88%	3.00%	0.88%	0.25%	0.25%	0.25%	0.49%	0.79%	0.34%	5.56%
	Rem8	0.78%	0.84%	2.04%	1.88%	2.35%	10.45%	7.84%	31.31%	20.75%	5.12%	2.46%	0.78%	0.26%	1.05%	2.93%	0.42%	8.73%
50	Rem9	0.42%	0.36%	0.39%	0.81%	0.29%	0.71%	0.58%	1.56%	5.75%	18.90%	39.75%	13.25%	0.91%	2.34%	4.77%	2.24%	6.95%
	Rem10	1.27%	0.39%	0.52%	0.80%	0.17%	0.44%	0.28%	0.44%	1.24%	4.40%	16.45%	44.10%	2.48%	4.32%	2.39%	8.36%	11.97%
Constitutive Multiple	Rem11	4.41%	7.29%	18.01%	2.31%	11.35%	4.48%	27.75%	8.97%	1.89%	0.77%	0.70%	0.35%	0.63%	1.19%	1.12%	0.77%	7.99%
	Rem12	0.63%	0.63%	0.54%	1.45%	0.91%	5.02%	1.22%	6.16%	26.84%	24.94%	12.00%	3.35%	0.41%	0.81%	8.78%	1.09%	5.21%
	Rem13	5.68%	2.41%	1.21%	2.07%	0.57%	0.69%	0.34%	0.86%	1.32%	2.58%	4.25%	15.33%	14.98%	7.41%	1.49%	23.71%	15.10%
	Rem14	1.11%	2.13%	1.19%	4.51%	1.28%	4.25%	1.28%	2.30%	6.04%	8.50%	17.60%	14.29%	2.21%	1.70%	12.76%	7.48%	11.39%

Figure S5. Distribution of probesets in each RL4452 embryonic module across the AC Domain modules. The number of probesets commonly expressed in each of the RL4452 embryo module (Rem1-14) and the AC Domain embryo modules (Aem1-16) is calculated as a percentage of the total number of probesets expressed in a given RL4452 embryo module. The gradient of the red color in the fill represents change in percentage. N/A indicates the percentage of probesets in a given RL4452 embryonic module with no expression in AC Domain embryo.
