# Supplementary material for: Seed maturation associated transcriptional programs and regulatory networks underlying genotypic difference in seed dormancy and size/weight in wheat (Triticum aestivum L.)
Source: BMC Plant Biol. 2017 Sep 16;17:154. doi: 10.1186/s12870-017-1104-5 (PMC5603048; doi:10.1186/s12870-017-1104-5)
Supplement: Supplementary file 6 — Fig. S6. Distribution of probesets in each RL4452 endospermic cluster across the AC Domain clusters. (PDF 416 kb) [file 12870_2017_1104_MOESM6_ESM.pdf]

RL4452 Endosperm		AC Domain Endosperm 20				30	40			50		Constitutive Multiple						
		Aen1	Aen2	Aen3	Aen4	Aen5	Aen6	Aen7	Aen8	Aen9	Aen10	Aen11	Aen12	Aen13	Aen14	Aen15	Aen16	#N/A
20	Ren1	31.34%	10.18%	2.02%	5.49%	3.18%	1.30%	1.59%	1.73%	0.94%	1.44%	1.66%	2.53%	3.18%	6.06%	5.34%	1.59%	20.43%
	Ren2	19.41%	34.62%	5.39%	14.47%	5.79%	1.36%	0.57%	0.23%	0.23%	0.17%	0.45%	0.28%	1.53%	1.93%	0.40%	1.14%	12.03%
	Ren3	8.30%	40.31%	14.39%	22.55%	3.05%	1.56%	0.39%	0.26%	0.06%	0.00%	0.06%	0.00%	1.49%	0.52%	0.39%	0.39%	6.29%
30	Ren4	6.69%	16.34%	7.09%	22.93%	12.20%	2.85%	1.97%	0.49%	0.89%	0.39%	0.98%	0.89%	1.48%	1.87%	0.49%	3.35%	19.09%
	Ren5	4.08%	4.08%	3.34%	6.68%	7.98%	4.45%	4.08%	5.19%	2.97%	1.11%	6.31%	2.04%	1.48%	3.34%	2.41%	6.68%	33.77%
	Ren6	4.10%	14.24%	19.04%	13.54%	9.44%	12.00%	3.64%	0.85%	0.15%	0.39%	1.39%	0.15%	6.11%	1.63%	0.85%	0.77%	11.69%
40	Ren7	5.07%	5.77%	8.42%	4.52%	3.27%	14.81%	19.95%	4.68%	0.94%	1.56%	3.12%	1.25%	10.44%	3.12%	2.26%	0.70%	10.13%
	Ren8	8.67%	10.80%	19.03%	7.08%	3.54%	11.95%	8.14%	1.42%	0.18%	0.35%	1.06%	0.35%	12.30%	1.95%	1.15%	0.27%	11.77%
	Ren9	5.22%	3.05%	1.97%	0.89%	1.28%	4.33%	19.49%	16.54%	2.26%	5.22%	3.54%	1.97%	7.68%	3.15%	8.56%	1.08%	13.78%

RL4452 Endosperm		Aen1	Aen2	Aen3	Aen4	Aen5	Aen6	Aen7	Aen8	Aen9	Aen10	Aen11	Aen12	Aen13	Aen14	Aen15	Aen16	#N/A
50	Ren10	0.95%	0.26%	0.35%	0.26%	0.35%	1.21%	2.86%	11.88%	17.17%	23.59%	5.03%	7.81%	0.87%	3.90%	3.21%	2.34%	17.95%
	Ren11	1.15%	0.44%	0.16%	0.16%	0.60%	0.27%	3.01%	27.49%	7.94%	31.76%	1.81%	4.44%	0.99%	3.29%	6.57%	0.82%	9.09%
	Ren12	0.20%	0.06%	0.03%	0.09%	0.12%	0.00%	0.64%	6.45%	26.84%	47.18%	0.67%	5.61%	0.15%	0.84%	2.30%	0.35%	8.48%
	Ren13	0.45%	0.34%	0.17%	0.34%	0.48%	0.03%	0.93%	3.38%	37.08%	23.78%	1.38%	7.51%	0.31%	2.21%	2.65%	1.31%	17.64%
Constitutive Multiple	Ren14	5.92%	25.02%	28.83%	17.58%	4.02%	4.61%	1.05%	0.46%	0.13%	0.07%	0.33%	0.07%	4.08%	0.66%	0.39%	0.39%	6.39%
	Ren15	3.15%	1.19%	1.45%	1.36%	1.19%	2.04%	13.80%	25.30%	4.00%	9.88%	5.28%	4.09%	3.07%	5.54%	5.03%	2.04%	11.58%
	Ren16	14.89%	19.13%	11.14%	7.99%	3.03%	3.39%	4.12%	2.78%	0.61%	0.48%	0.97%	0.61%	10.41%	3.39%	3.27%	0.85%	12.95%
	Ren17	3.79%	2.23%	0.67%	1.79%	2.79%	0.78%	1.90%	3.24%	10.60%	5.47%	3.91%	7.92%	1.23%	8.26%	8.93%	7.03%	29.46%
	Ren18	5.28%	1.76%	0.70%	1.06%	1.06%	1.41%	4.10%	10.32%	9.61%	14.89%	2.11%	5.74%	2.23%	7.50%	16.65%	1.29%	14.30%

Figure S6. Distribution of probesets in each RL4452 endospermic module across the AC Domain modules. The number of probesets commonly expressed in each of the RL4452 endosperm module (Ren1-18) and the AC Domain endosperm modules (Aen1-16) is calculated as a percentage of the total number of probesets expressed in a given RL4452 endosperm module. The gradient of the red color in the fill represents change in percentage. N/A indicates the percentage of probesets in a given RL4452 endospermic module with no expression in AC Domain endosperm.
